# How social media and peer learning influence student-teacher self-directed learning in an online world under the ‘New Normal’

**DOI:** 10.1016/j.heliyon.2023.e13769

**Published:** 2023-02-16

**Authors:** Paitoon Pimdee, Attaporn Ridhikerd, Sangutai Moto, Surapong Siripongdee, Suwanna Bengthong

**Affiliations:** School of Industrial Education and Technology, King Mongkut's Institute of Technology Ladkrabang (KMITL), Bangkok, Thailand

**Keywords:** 21st-century abilities, Competencies, Learning desire, Pre-service teachers, Self-management, Thailand

## Abstract

The study aimed to investigate three aspects of Thai student-teacher self-directed learning (SDL) competency. These were the student-teachers opinions concerning their use of social media (SM), self-management (SM), and learning desire (LD). The sample group was 468 student-teachers enrolled in a Bachelor of Industrial Education Program at the King Mongkut's Institute of Technology Ladkrabang in Bangkok, Thailand, in the Academic Year 2021. The research instrument consisted of an SDL competency questionnaire whose discrimination (corrected item-total correlation) values were determined to be between 0.37 and 0.69, which also had a confidence level of 0.91. Data analysis used LISREL 9.10 for the study's second-order confirmatory factor analysis (CFA). Descriptive statistics analysis included the mean and standard deviation (SD), which was accomplished using IBM's® SPSS® for Windows Version 21. Three models were developed for the study. These included the *social media* (SM) model containing 285 participants, the *peer learning* (PL) model, which contained 183 participants, and the *total group* (TG) model, which contained everyone surveyed (*n* = 468). The final analysis from the second-order CFAs showed that student-teacher SDL competency for self-control (SC) (0.96) was valued most by the student-teachers. However, their learning desire (LD) (0.87) and self-management (SM) (0.80) skills were somewhat behind. Moreover, in the Pearson Product Moment Correlation (PPMC) (*r*) analysis of the 24 variable relationships, the strongest was related to each student-teacher's learning desire. However, the weakest variable relationship was related to their ability to set high personal standards and the self-discipline to achieve them. Finally, quite interestingly, 60.90% of the student-teachers indicated that their SDL is gotten from social media (SM) resources compared to learning from their peers (PL) around them.

## Highlights

• Student-teacher self-directed learning aspects were investigated.
• The aspects included social media, self-management, and learning desire.
• The sample contained 485 Thai student-teachers.
• The strongest relationship between the 24 variables was learning desire.
• 60.90% indicated self-directed learning is obtained from social media.

## 1 Introduction

In 2015 the United Nations released a fifteen-year vision of Sustainable Development Goals (SDGs) that included seventeen main goals and 169 targets [1,2], which were stated as being essential for the planet and humanity. Specific to education was the UN's SDG Goal 4, which articulated promoting equality in education. It also set objectives for inclusive, lifelong education promotion opportunities for all global citizens and self-directed learning (SDL) ability [3]. However, SDL is nothing new and can be traced back to Greek philosophers such as Aristotle, Socrates, and Plato [4,5].

However, information and communication technologies (ICT) have radically changed how SDL is accomplished and delivered in formal and informal education [6,7]. As such, SDL has become an increasingly essential learning pedagogy in the 21st century. This has necessitated a shift from teacher-directed 'chalk and talk’ environments to one where the student becomes the learning process center [[8], [9], [10]].

Although it is difficult to imagine how SDL would be discussed today without online learning, the merger of the two has seemingly accelerated since the beginning of the worldwide COVID-19 pandemic in early 2020. Along with shuttering global trade, tourism, and traditional classroom education [11], educators and civic leaders have sought ways to educate without exposing teachers and students to the ravages of the pandemic [12,13]. One common method has been the jump to online education and digital devices.

Moreover, Eschenbacher and Fleming [14] reviewed transformative learning and lifelong learning since the COVID-19 crisis began and reported that there had been significant damage done to the stakeholders. This includes damage to the workers, families, individuals, and economies since its inception. The authors also stated that this has led to an unprecedented and urgent search for knowledge and suggest that learning is the only method available to find our way out of this terrible predicament. Finally, quite interestingly, they state that *not-knowing* has become the *New Normal*.

In Thailand, when traditional classroom education ceased due to the pandemic's forced lockouts, Thailand's Ministry of Education (MOE), as early as April 21, 2020, began propagating the idea of a *New Normal* in education. In this context, the New Normal included online education, Distance Learning Television (DLTV), ICT development, and digital learning tools such as smartphones [[15], [16], [17]]. Additionally, the MOE started reallocating funds from traditional schools and classrooms to online learning communities and course development.

Quite interesting, the ministry detailed how 'online learning’ would be differently applied according to the student grade level. Thus, Thai Grades 1–6 would use one-way communication for lesson delivery, while Grades 7–12 would use two-way communication methods focused on student/teacher interaction.

However, it was implied that although it was recognized that many teachers needed to gain experience with online teaching, it would be the responsibility of the teachers to rehearse and practice it before they use it on their students. Responsibility for using the online resources and courses was then placed on the teachers and, to some degree, the parents, who were now expected to function as assistant teachers. This became Thailand's '*New Normal*’ when applied to education [[18], [19], [20]].

To accomplish this *New Norma,* Thailand quickly moved education into an online world. This has taken various forms depending on Internet availability, speed, costs, and technical support to each institution, staff, and students. Even social media commercial platforms such as Line and Facebook are being used now to support these broad educational objectives [21,22]. However, ‘online education’ is nothing new and has been examined, evaluated, and used as early as 1960, with terminal computer networks even before the ‘invention’ of the Internet and personal computers [20].

One student-centered digital learning environment which has gained significant fame over recent years is the ‘flipped classroom’ [[23], [24], [25], [26]]. According to the recognized early implementers of this online and highly flexible learning pedagogy, it allows students to choose when, where, and the pace of their learning [27, 28]. It also allows students to master the content and provide a method by which they can execute SDL effectively [29]. Therefore, well-developed SDL online environments allow students to take better control over their learning. This is consistent with Rashid and Asghar [30], who also reported that ICT use in an SDL environment positively correlates to student engagement and academic success.

Therefore, the authors will expand the discussion in Part 2's literature review from the brief introduction. In part 3, the study's methods are detailed. In Part 4, the results are from analyzing the 3 s-order CFA models for the study's 468 student-teacher respondents [15]. In Part 5, a Discussion is presented. Finally, throughout the paper, analysis is brought forth on how today's *social media* vs. *peer learning* affects a student-teacher's *self-management* (SM), *self-control* (SC), and *learning desire* (LD) as components of their SDL process. The study hopes to give valuable input into online learning under the '*New Normal*’ [12,13].

## 2 Literature review

### 2.1 21st-century learner performance

A highly recognized list of 21st-century competencies in child development is outlined by Singapore's Ministry of Education [31]. Their discussion indicates competency requirements are necessary due to technological advancements, ever-changing demographics, and globalization.

Moreover, the Singapore Ministry of Education's (MOE) 'core values' include student self-awareness, self-management, decision-making responsibility, social awareness, and managing their relationships. Also, students should be self-directed learners (SDLs), confident, concerned citizens, and active contributors.

These social-emotional competencies are necessary skills that learners need to develop for healthy identities and emotions. There is also the additional need to develop sensitivity, responsibility, care, and concern for others. Finally, social-emotional competency skills relate to how relationships are made positive, how challenges are handled, how responsible decisions are made, and how an individual acts for the good of self, others, and society.

These ideas are also consistent with multiple scholars who have suggested that there needs to be a focus on the rising need for contemporary skills. These have been identified as creativity, curiosity, critical thinking, entrepreneurship, collaboration, communication, growth mindset [[32], [33], [34]], and global competence [35,36]. Similarly, these ideas support a higher education institution survey from the World Economic Forum that reported what employers would be looking for in new hires in 2020. These included the ability for creativity, critical thinking, complex problem-solving, emotional intelligence (EI), and teamwork [37]. Lee and Yuan [38] also found support for these ideas in China due to the government's significant push to create an innovative culture and entrepreneurship through innovation education.

Manning [39] has also claimed that the relationship between SDL and lifelong learning is reciprocal, with SDL viewed as an approach to pursuing learning throughout one's life. Moreover, lifelong learning empowers students with multiple skills and competencies required to pursue self-education after they complete their formal education. Also, SDL assists learners in information comprehension while serving as a mechanism for lifelong learning [40].

### 2.2 Social-media learning (SML)

Ever-increasing research has indicated the importance of *social-media learning* in self-directed learning. In one study concerning Australian higher education, Kent [41] reported that when Facebook was used to supplement a course's online learning management system (LMS) and online teaching, the social media platform significantly enhanced learner activity levels in discussion forums. This is consistent with Mentz et al. [5], who discussed open educational resources (OERs) and identified social media as an essential tool in SDL. In another study on collaborative learning for 21st-century learning, the authors emphasized social media usefulness in higher education [42].

However, as numerous authors have pointed out, more is needed with digital learning resource access if online learning is to be successful. In Thailand, even before the COVID-19 pandemic, multiple studies were undertaken on how to implement educational change. Numerous problems were then detailed regarding how ICT, the Internet, and online learning faced many challenges, even on their best days. One common theme in these studies was the need for educators and administrators to keep pace with change and new situations better. Other problems included some teachers’ unwillingness to change from traditional teaching concepts and their inability to embrace new knowledge and skills. Other problematic themes also included how well or poorly teachers could develop online teaching materials, which then was connected to their ability at course management and evaluation guidelines.

Admittedly, COVID-19 has forced many teachers to seek more training, often in their own free time and at their own expense [17]. Another problem in Thailand, even before the pandemic forced education online, was that many teachers and student teachers were teaching subjects they needed to gain experience or qualifications. In addition, teachers often complain that they are forced to take on other tasks besides teaching, which affects their potential and professional competency standards. As a result of these problems, Thailand's educational competitiveness, compared to other countries, has decreased in recent years [43].

However, not all the problems are due to the educators but instead due to the limits in educational budgets [44], poor or no training [17], poor information communication technology (ICT) infrastructure, limited or no Internet connections, or lack of knowledgeable ICT support staff [45]. Other studies have explicitly identified how rural schools operate at a significant disadvantage to urban schools due to their lack of ICT capabilities, including software, computers, and consistent broadband Internet connectivity [[46], [47], [48]].

Added to this difficulty and complexity, UNESCO has also specifically pointed out that a school's core mission is promoting a student's well-being which is best served by a structured setting in which children can develop and learn social competencies [49]. The report implies that online teaching and professional learning communities (PLCs) must meet these obligations. Therefore, solutions are needed, and they are needed quickly.

### 2.3 Peer learning (PL.)

Multiple authors have suggested that various forms of peer, collaborative, or cooperative learning should be used within higher education courses to assist learners' outcomes [50]. It has also been pointed out that ICT now okays a significant role in peer learning success and what methods can be used [51].

In a supporting study on how PL affects undergraduate students in an architectural sustainability course, the authors reported that PL was very effective when other students acted as tutors. This then increased each student's commitment, motivation, and knowledge, while simultaneously increasing their final scores 25% higher than the students' previous classes [52].

Although peer learning's benefits have long been recognized and are especially relevant today, others find the 'devil in the detail’ as poor assessment processes. This is true for Boud et al. [50], who detailed how current assessment practices undermine PL goals, leading to student rejection of PL. One problem mentioned is that some assessment processes give students the message that collaborative efforts are cheating and that individual achievement is preferred.

However, other studies have noted that students gained psychological well-being after four years of university study using PL. PL also significantly and positively affected most of the Ryff well-being subscales (purpose in life, environmental mastery, positive relations with others, autonomy, personal growth, and self-acceptance) [53,54]. This is consistent with research in Thailand which stated that PL is highly effective in improving a student's motivation, learning, and friendships [55].

### 2.4 Self-directed learning (SDL)

Frequently, SDL is viewed as a way in which individual initiative allows learners to analyze their learning needs, with or without external support. This includes the formulation of learning goals, the identification of personal and material resources, the selection and implementation of meaningful learning strategies, and the ability to self-evaluation of learning outcomes [56].

As previously mentioned, the conceptualization of SDL goes back multiple millenniums [4,5]. However, SDL has become a subject of contemporary research over the past several decades [5,[57], [58], [59], [60]], with learning pedagogies like flipped learning emphasizing the learner's freedom to choose what, when, where, and how they learn and whether the learning is mastered [61,62].

According to Long [63], SDL embraces three psychological characteristics. These include the ability for self-regulation, motivation, and metacognition. There are also four secondary psychological dimensions. These include confidence, control, choice, and competence.

Capability theory also suggests that the learning process has three pillars. These include self-directedness, self-regulation, and self-determination, which have been stated as fundamental to other approaches or theories. As such, learners should value these capabilities if they wish for them to be developed and fulfilled [64].

Brockett & Hiemstra [4] have also deﬁned SDL as a learning process in which students plan, evaluate, establish goals, and select and seek resources. Barnes et al. [65] also felt that today's ‘Net Generation’ can use SDL processes to achieve better opportunities. This is accomplished by establishing interactive environments, feedback channels, and assignment choices that allow different and meaningful learning resources and experiences.

Other scholars have investigated SDLs' use in a team-based learning style of learner-determined needs and goals [66,67]. Using team-based learning, teachers can create flexible learning paths allowing learners to select their own learning components determined by their interests and needs.

Additionally, SDL is connected to personalized learning which today is often combined with *competency-based education* (CBE). This allows non-traditional higher education to serve graduates’ employer needs [68,69].

These ideas are consistent with Zhao and Watterston [11], who saw the need for three significant educational changes as the world hopefully pulls out of the COVID-19 pandemic chaos. These included curriculums that are developmental, personalized, and evolving. Pedagogies should also be authentic, inquiry-based, student-centered, and purposeful. The instruction delivery also needs to capitalize on synchronous and asynchronous learning strengths.

### 2.5 Research objectives

1) To analyze the students' SDL competency components, classified by learning styles and the total sample of student-teachers.
2) To study the level of opinions about the student-teachers SDL competence, classified by learning styles and the total sample of student-teachers.

## 3 Materials and methods

Before the study's participation of student-teachers and experts, the authors sought permission from their university's Human Research Ethics Committee. After consultation, permission was granted for the study. Every participant was then given a form in which the objectives were explained, which assured each individual that their data privacy and participation were assured. The participants were also told they could drop out of the study if they desired. All collected forms from the participants were signed and secured in a locked faculty filing cabinet.

### 3.1 Population and sample

The population for the study consisted of 1749 undergraduate students in a Bachelor of Industrial Education program in the Academic Year 2021 at the authors' university. Sample size determination was established from recommendations from multiple scholars who have reported that the sample sizes should be approximately 10–20 times the model's observed variables [[70], [71], [72]]. As the survey instrument used 24 observed variables, a multiple of 20 was used to assure better survey reliability (24 × 20 = 480). This was then rounded upwards to 500 as a sample target. Simple random sampling was combined with a student lottery system for each field of study until the number of participants was achieved according to the previously determined proportion (Table 1).

**Table 1:** The target and collected sample groups were classified by field of study.

Programs	Population	Samples		
		Target	Collected	
			Number	%
Agricultural Education	350	100	98	97.94
Engineering Education	528	151	147	97.39
Architecture	259	74	68	91.84
Design Education	304	87	84	96.66
Interior-Environment Design	308	88	71	80.64
**Totals**	**1749**	**500**	**468**	**93.60**

### 3.2 Research instrument

The research instrument contained a background section (Part 1) and a questionnaire to measure the students' self-learning competency (Parts 2–4). Part 1's items were related to each student-teacher's gender, their field of study, cumulative GPA, and self-learning resources. Part 2's eight items were related to each individual's self-management (SM) skills. Part 3's eight items concerned their self-control (SC) skills. Finally, Part 4's eight items probed each student-teacher's learning desire (LD).

After questionnaire development, the instrument was evaluated by a panel of experts, from which items were adjusted for better clarity. After this, the researchers used the questionnaire to conduct a pilot test with 30 similar undergraduate students enrolled in the same program whose input was not used in the subsequent study. Analysis used a 5-level estimation scale for Part 2–4's 24 items. Their questionnaire responses were then analyzed for discrimination reliability using Cronbach's Alpha (α) coefficient method and a corrected item-total correlation method (Table 2).

**Table 2:** Total aspect items, the power of discrimination, and the confidence of the questionnaire were classified by aspects.

Aspects	Items	Discrimination	(α)
Self-management (SM)	8	0.42–0.76	0.82
Self-control (SC)	8	0.25–0.69	0.81
Learning desire (LD)	8	0.41–0.63	0.83
Overview	24	0.37–0.69	0.91

Note. The overview shows the results from all 24 items analyzed from the study's final second-order CFA. This resulted in discrimination and α values being higher for each of the three aspect's eight observed variables. Therefore, α values and discrimination values were lower when analyzed individually.

### 3.3 Data collection

The researchers collected data from April 2021 to June 2021 with a sample of undergraduate student-teachers enrolled in the *Bachelor of Industrial Education Program* at Thailand's King Mongkut's Institute of Technology Ladkrabang in the academic year 2021. Initially, 500 students were targeted for participation in a survey using *Google Form*s [73]. A team of research assistants coordinated with a network of faculty advisors and student-teachers in each discipline and level. After this, 468 complete questionnaires were returned from this effort, representing 93.60% of the target sample. The size of the sample collected was considered sufficient and was consistent with sample size recommendations from multiple scholars who have reported that the sample sizes should be approximately 10–20 times the model's observed variables [[70], [71], [72]].

### 3.4 Data analysis

A second-order CFA using LISREL 9.10 was performed on each student-teacher's responses concerning their SDL competency. The student teacher's SDL sources used two primary resources in the three models. These were their *peers* and *social media*. This data was then interpreted for validity using a second-order CFA for the opinions on the student-teachers SDL competency using the harmonization index criteria partially selected from Refs. [74,75]. IBM's® SPSS® for Windows Version 21 program was used for the descriptive statistics analysis with the analysis interpretation using mean $\bar{}$ scores and standard deviation (SD) scores. Mean score evaluation criteria and interpretation used a five-level Likert-type opinion scale in which 5 was strongly agree (4.50–5.00), 4 was agree (3.50–4.49), 3 was unsure (2.50–3.49), 2 was disagree (1.50–2.49), and 1 was strongly disagree (1.00–1.49).

## 4 Results and discussion

### 4.1 Student-teacher demographics

The responses from each student-teacher concerning their demographics are detailed in Table 3. First, the authors noted the imbalance between females (60.04%) and males (39.96%) student-teachers. This is now common in many education studies which report similar education enrollment gender imbalances in Southeast Asia. This is especially acute in Thailand and Malaysia, as females make up more than 60% of Thai universities and 64% of Malaysian university enrolments [[76], [77], [78]]. Second, most student-teachers (48.08%) maintained a GPA range of 3.00–3.49 during the COVID-19 online learning *'New Normal*.’ Finally, quite interestingly, 60.90% of the student-teachers indicated that their SDL is gotten from *social media* resources compared to learning from their *peers* around them (39.10%).

**Table 3:** Student-teacher general information (*n* = 468).

Variables	Category	Number	%
Gender	Male	187	39.96
	Female	281	60.04
Academic Program	Agricultural Education	98	20.94
	Engineering Education	147	31.41
	Architecture Education	68	14.53
	Design Education	84	17.95
	Interior-Environment Design Education	71	15.17
Current Cumulative GPA	2.00–2.49	31	6.62
	2.50–2.99	133	28.42
	3.00–3.49	225	48.08
	3.50–4.00	79	16.88
Self-directed learning resources	Learning from the people around you (peer learning)	183	39.10
	Learning from social media	285	60.90

### 4.2 Student-teacher general characteristics

The results shown in Table 4 details the students' overall opinions about their SDL competency using a descriptive statistics analysis with IBM's® SPSS® for Windows Version 21 software. The mean scores from highest to lowest are *self-control* (mean = 4.10), *learning desire* (mean = 4.07), and *self-management* (mean = 3.87), respectively.

**Table 4:** Student-teacher opinion levels concerning their self-learning styles in each aspect.

Aspects	Learning Styles						Total		
	Peers (PL)			Social Media (SM.)					
	Mean	SD	Level	Mean	SD	Level	Mean	SD	Level
Self-management (SM)	3.85	.55	High	3.88	.52	High	3.87	.53	High
Self-control (SC)	4.09	.56	High	4.10	.49	High	4.10	.52	High
Learning desire (LD)	4.05	.56	High	4.09	.54	High	4.07	.55	High
Total	3.99	.48	High	4.02	.45	High	4.01	.46	High

*Note*. SD = standard deviation.

### 4.3 Model analysis

Table 5 shows the detailed analysis and results for the study's three primary aspects and 24 observed variables. As such, when considering the components classified by model, it was found that the total group (TG) model found that *component weights* (β) were positive between 0.80 and 0.96 and differed from zero at the 0.01 level for all components. The order of importance was based on the β, with *self-control* (0.96) first, *desire for learning* (0.87) second, and *self-management* (0.80) ranked third in importance, respectively.

**Table 5:** Element weight values for the Peer Learning (PL) Model, the Social Media (SM) Model and the Total Group Model (TG) and their three aspects of SM, SC, and LD.

Groups									
Aspects/Variables	Learning style						Total Group		
	Peer Learning			Social Media					
	β	t	R^2^	β	t	R^2^	β	t	R^2^
**Self-management (SM)**	**.82**	**8.28**	**.67**	**.79**	**9.90**	**.62**	**.80**	**13.08**	**.65**
1. I manage my time well (a1).	.66	↔	.44	.66	↔	.44	.67	↔	.46
2. I am self-disciplined (a2).	.64	9.24	.41	.59	11.40	.34	.63	14.82	.40
3. I can manage my time (a3).	.69	8.55	.48	.67	9.69	.45	.66	12.43	.43
4. I am serious about using my time (a4).	.66	8.07	.44	.55	8.27	.31	.62	11.64	.39
5. I have good organizational skills (a5).	.82	9.50	.67	.73	10.42	.54	.74	13.72	.55
6. I learn systematically (a6).	.78	9.17	.61	.69	9.84	.48	.75	13.92	.56
7. I use planning to solve problems (a7).	.66	7.94	.43	.63	9.17	.40	.64	12.30	.41
8. I set priorities in my work (a8).	.68	8.35	.46	.63	9.14	.40	.64	12.29	.41
**Self-control (SC.)**	**.93**	**8.24**	**.87**	**.99**	**8.06**	**.99**	**.96**	**11.20**	**.92**
9. I am in control of my personal life (a9).	.62	↔	.39	.49	↔	.24	.54	↔	.29
10. I have high personal standards (a10).	.62	7.10	.38	.43	5.76	.19	.50	8.55	.25
11. I always set my own learning goals (a11).	.71	7.92	.51	.68	7.47	.47	.69	10.47	.48
12. I evaluate my performance (a12).	.75	8.23	.56	.70	7.36	.50	.71	10.26	.51
13. I take responsibility for what I do (a13).	.69	8.84	.48	.58	7.70	.34	.60	11.15	.36
14. I focus on problems (a14).	.78	7.80	.61	.73	7.76	.54	.74	10.85	.55
15. I am aware of my imitations (a15).	.63	7.08	.40	.52	7.09	.28	.53	9.77	.28
16. I can research information for my use (a16).	.73	8.05	.54	.66	7.38	.43	.70	10.57	.49
**Learning desire (LD.)**	**.81**	**8.76**	**.66**	**.89**	**9.89**	**.79**	**.87**	**13.67**	**.76**
17. I always want to learn new things (a17).	.73	↔	.53	.61	↔	.38	.68	↔	.49
18. I have a learning desire (a18).	.76	11.60	.58	.68	14.98	.46	.73	18.88	.53
19. I like challenges (a19).	.64	7.96	.41	.59	9.99	.35	.62	12.52	.38
20. I enjoy learning (a20).	.64	8.00	.41	.73	9.71	.54	.69	12.86	.48
21. I can critically evaluate new ideas (a21).	.70	8.74	.49	.76	9.93	.57	.65	12.19	.43
22. I like to gather facts before making decisions (a22).	.69	8.66	.48	.70	9.71	.49	.71	12.89	.50
23. I like to evaluate what others do (a23).	.62	7.64	.38	.68	9.47	.46	.65	11.87	.43
24. I am open to new ideas (a24).	.69	8.45	.47	.68	9.20	.46	.68	12.54	.46

*Note*. β = component weights, t = t-value, R^2^ = coefficient of determination.

The model which evaluated peer learning was found to have positive element weights (β) between 0.81 and 0.93 and differed from zero at the 0.01 level for all components. The order of importance was based on the β with *self-control* (0.93) first, *self-management* (0.82) second, and *learning desire* (0.81) third in importance, respectively.

The group model for social media learning was found to have positive element weights (β) between 0.79 and 0.99 and differed from zero at the 0.01 level for all components. The order of importance was based on the β with *self-control* (0.99) first, *learning desire* (0.89) second, and *self-management* (0.79) third in importance, respectively.

Furthermore, the *t*-test analysis used the criteria of |t|≥1.96 to confirm the t-value significance [79]. Therefore, from the results in Table 5, the lowest reported t-value for the three aspects and 24 observed variables was a10 (5.76, *p* ≤ .01), indicating significant support for the study's second-order CFA model. Testing of the coefficient of determination (R2) used values established by Hooper et al. [80] for evaluation, with R^2^ values suggested as not being ≤0.20. This was met for all but one variable (0.19). Additionally, it was determined that the three components of the students' self-learning competency had high construct reliability (CR ≥ 0.60).

### 4.4 Pearson product-moment correlation (PPMC) (r) analysis of SDL competency

Table 6 shows the relationship results between the 24 observed variables' correlation coefficients using a PPMC (*r*) analysis. Results for the 24-indicator testing of PPMC *r* construct validity (CV) showed that all the final model's variables were correlated and in the same direction. Also, the interpretation of this data used values suggested by Akoglu [81], in which *r* value strength interrelationship interpretation is suggested as 0.10–0.29 (weak), 0.30–0.49 (moderate), and 0.50–1.00 (strong). Therefore, *a18* to *a17* was the strongest (0.74), related to each student-teacher's learning desire. However, the weakest was *a10* to *a2* (0.16), related to high personal standards and the self-discipline to achieve them.

**Table 6:** Correlation coefficient (r) relationship analysis.

Var	a1	a2	a3	a4	a5	a6	a7	a8	a9	a10	a11	a12	a13	a14	a15	a16	a17	a18	a19	a20	a21	a22	a23	a24
a1	1.00																							
a2	.62	1.00																						
a3	.46	.43	1.00																					
a4	.40	.40	.41	1.00																				
a5	.51	.45	.56	.39	1.00																			
a6	.52	.53	.48	.40	.56	1.00																		
a7	.41	.35	.43	.40	.46	.51	1.00																	
a8	.42	.42	.30	.41	.45	.45	.52	1.00																
a9	.26	.19	.18	.27	.27	.30	.33	.37	1.00															
a10	.27	.16	.36	.25	.34	.34	.34	.20	.28	1.00														
a11	.35	.33	.39	.31	.41	.40	.40	.29	.35	.48	1.00													
a12	.35	.36	.37	.29	.44	.41	.35	.33	.32	.39	.65	1.00												
a13	.27	.27	.20	.28	.29	.34	.31	.45	.50	.25	.40	.43	1.00											
a14	.39	.34	.35	.33	.46	.48	.38	.37	.38	.34	.52	.53	.52	1.00										
a15	.22	.18	.23	.23	.26	.26	.24	.33	.42	.26	.39	.39	.49	.53	1.00									
a16	.34	.35	.34	.27	.43	.42	.34	.35	.37	.38	.47	.50	.42	.52	.37	1.00								
a17	.29	.29	.34	.34	.33	.33	.28	.34	.32	.25	.41	.37	.38	.37	.31	.41	1.00							
a18	.32	.29	.30	.34	.29	.34	.33	.34	.38	.25	.46	.39	.42	.44	.32	.45	.74	1.00						
a19	.32	.26	.28	.27	.26	.32	.25	.26	.28	.30	.36	.40	.31	.37	.28	.36	.52	.55	1.00					
a20	.36	.42	.37	.35	.39	.38	.30	.27	.29	.25	.37	.42	.30	.44	.27	.39	.47	.52	.52	1.00				
a21	.36	.30	.41	.28	.46	.40	.32	.27	.25	.26	.46	.44	.26	.43	.24	.40	.44	.47	.54	.62	1.00			
a22	.30	.31	.32	.38	.35	.39	.39	.43	.37	.27	.42	.41	.41	.41	.35	.41	.48	.48	.40	.47	.53	1.00		
a23	.32	.29	.31	.22	.32	.31	.29	.36	.33	.23	.39	.47	.36	.45	.36	.39	.37	.44	.39	.43	.51	.57	1.00	
a24	.30	.26	.22	.27	.31	.31	.31	.44	.39	.17	.33	.36	.49	.41	.42	.37	.50	.51	.42	.33	.40	.55	.47	1.00
Skew	−.48	−.69	−.75	−.45	−.52	−.33	−.77	−.52	−.89	−.67	−.77	−.79	−.52	−.46	−.35	−.43	−.55	−.56	−.48	−.69	−.75	−.45	−.52	−.33
Kurt	.44	1.06	.67	−.06	.50	.04	.94	.71	2.03	.51	1.36	1.53	−.10	.08	.20	.50	.62	.30	.44	1.06	.67	−.06	.50	.04

Also, when we examine the LISREL 9.10 skewness and kurtosis values, it is easier to interpret the results by knowing that the suggested values for skewness should be ≤ |2| and kurtosis ≤ |7| [82,83]. Therefore, all relationship values were statistically significant as they were between 0.16 and 0.74. Table 6 also details the observed variables' skewness values (−0.89 to −0.33) (≤|2|) and kurtosis values (2.03–0.04) (≤|7|). Finally, all relationships were significant at the *p* ≤ .01 level.

### 4.5 Goodness-of-fit (GoF) analysis

Table 7 shows the GoF analysis for the student-teacher peer, social media, and total group SDL second-order CFA models. The results suggest that the models were consistent with the empirical data.

**Table 7:** Criteria, theory, and results for the three model GOF appraisals.

Criteria Index	Criteria	Three Models GoF Values			Theory Support
		Peers	Social media	Total Group	
Chi-square: χ2	*p* ≥ .05	0.97	0.92	0.93	[74, 75]
Relative Chi-square: χ2/df	≤2.00	0.82	0.86	0.85	[84]
RMSEA	≤0.05	0.00	0.00	0.00	[80]
GFI	≥0.90	0.93	0.96	0.97	[85]
AGFI	≥0.90	0.90	0.93	0.96	[80]
RMR	≤0.05	0.04	0.03	0.02	[80]
SRMR	≤0.05	0.04	0.03	0.02	[80]
NFI	≥0.90	0.98	0.99	0.99	[72]
CFI	≥0.90	1.00	1.00	1.00	[72]

### 4.6 Social media model second-order CFA results (n = 285)

**Fig. 1:**
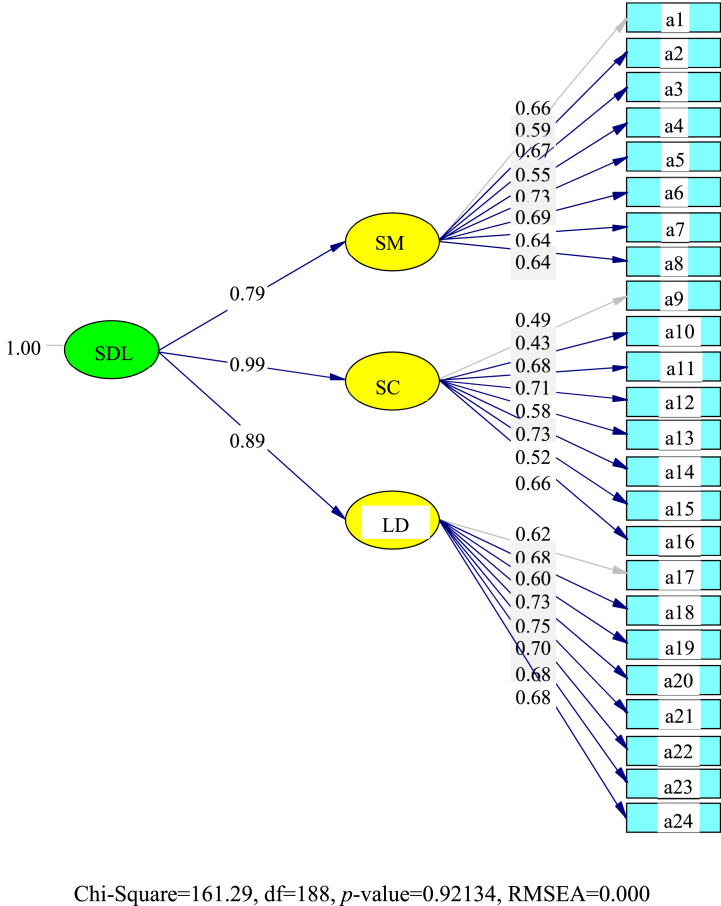
The final student-teacher second-order CFA for social media use in self-directed learning (SDL). **Note.** a1 – a24 are described in Table 5.

The final student-teacher second-order CFA for SDL competency for social media use is shown in Fig. 1. It was determined that self-control (0.99) was valued most by the student-teachers. Their learning desire (0.89) and self-management (0.79) skills were somewhat behind.

### 4.7 Peer learning model second-order CFA results (n = 183)

**Fig. 2:**
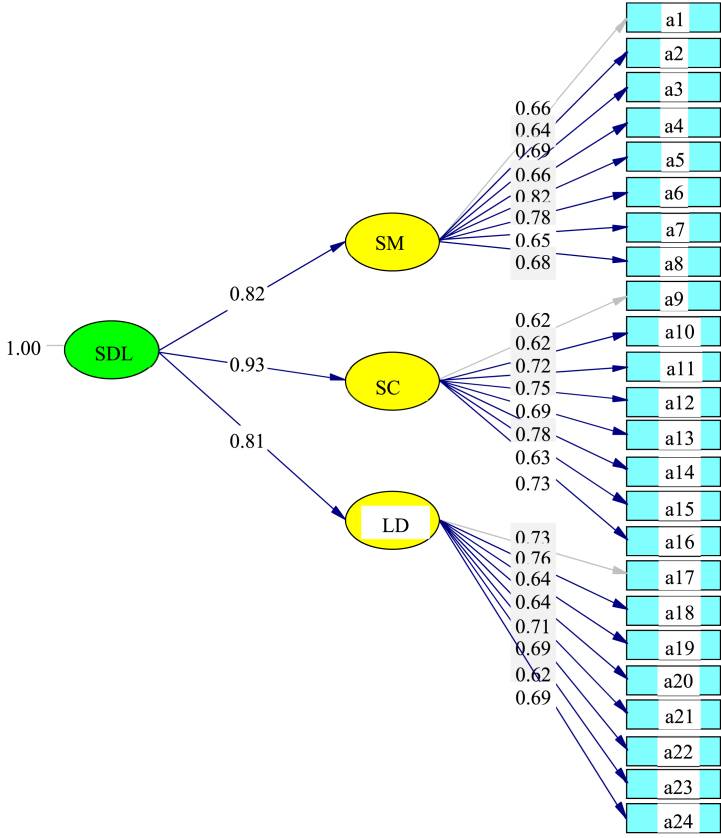
The final student-teacher second-order CFA for peer learning for self-directed learning (SDL). **Note.** a1 – a24 are described in Table 5.

The final student-teacher second-order CFA for SDL competency for peer learning use is shown in Fig. 2. It was determined that self-control (0.93) was valued most by the student-teachers. Their self-management (0.82) skills and learning desire (0.81) were second and third, respectively.

### 4.8 Total group model second-order CFA results (n = 468)

**Fig. 3:**
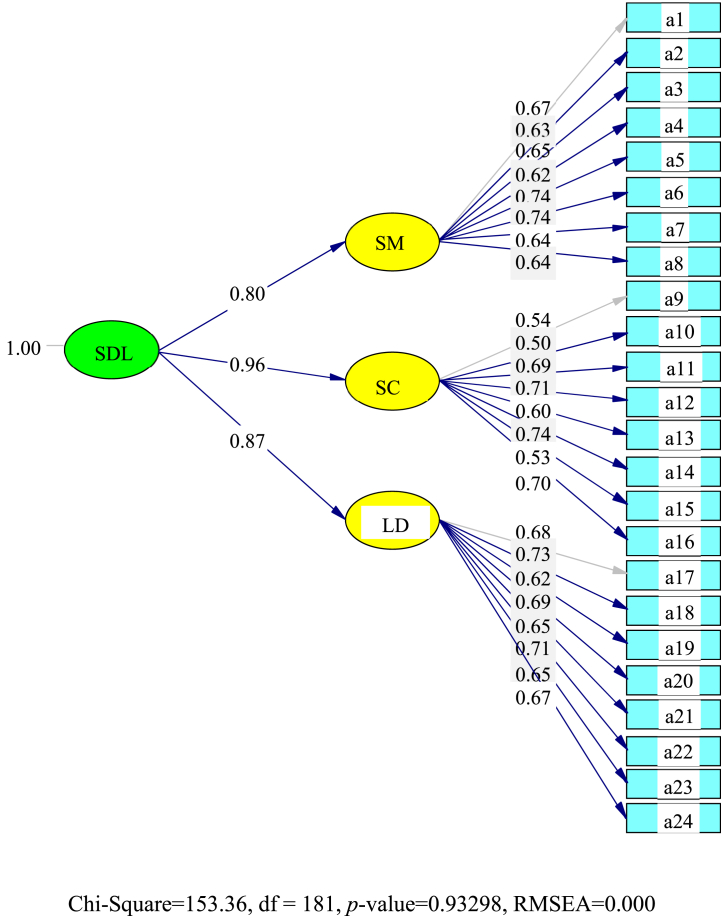
The final student-teacher second-order CFA for the total group (TG) for self-directed learning (SDL).**Note.** a1 – a24 are described in Table 5.

The final student-teacher second-order CFA for SDL competency from the total group (TG) is shown in Fig. 3. It was determined that self-control (0.96) was valued most by the student-teachers. This was followed by their learning desire (0.80) and self-management (0.80) skills being somewhat behind.

### 4.9 Second-order CFA CR and AVE results

Table 8 shows the composite reliability (CR) and average variance extracted (AVE) values for each SDL student-teacher aspect. Although all values for CR are ≥0.7 and are acceptable [86], the values for the AVE are seemingly low (≤0.5). However, Fornell and Larcker [87] have suggested that when the CRs are ≥0.6 and the AVEs are ≥0.5, values as low as 0.4 can be accepted. It should be noted that when all three aspects were analyzed together (24 variables) within the final second-order CFA, the AVE values were all higher, resulting in all AVE values being significantly higher than 0.50.

**Table 8:** The CR and AVE of each of the three student-teacher SDL groups.

Aspects	Models					
	PL (*n* = 183)		SM (*n* = 285)		TG (*n* = 468)	
	CR	AVE	CR	AVE	CR	AVE
Self-management (SM)	0.89	0.49	0.85	0.42	0.87	0.45
Self-control (SC)	0.88	0.48	0.82	0.37	0.84	0.40
Learning desire (LD.)	0.88	0.47	0.87	0.46	0.87	0.46
**Total**	**0.89**	**0.73**	**0.92**	**0.80**	**0.91**	**0.77**

## 5 Discussion

The Thai student-teacher self-directed learning (SDL) competency involved three sample groups and models. They were the group learning from social media (SM) (*n* = 285), the group learning from people around them (PL) (*n* = 183), and a model for the total group (TG) of student-teachers (*n* = 468). Moreover, these three groups were evaluated using three primary components. These included self-management, self-control, and learning desire.

Results revealed that each of the three models' second-order CFAs was consistent with the empirical data, the component weights were positive, and the order of influence weight was similar in all three models. These study findings were also consistent with ideas from Knowles [56], who reported that SDL consists of targeted learning, identification of personal and material resources, choosing and implementing meaningful learning strategies, and the ability to evaluate self-learning. These concepts also agree with Long [63], who said that SDL captures three psychological characteristics. These include self-control, motivation, and metacognition. There are also four secondary psychological dimensions. These include confidence, control, choice, and competence.

### 5.1 Social media model second-order CFA results discussion (n = 285)

The final student-teacher second-order CFA for SDL, competency from social media use, determined that self-control (0.99) was valued most by the student-teachers. However, their learning desire (0.89) and self-management (0.79) skills were somewhat behind.

These findings concerning the importance of social media and technology's use under the *New Normal* in SDL are reinforced by several studies. These include Lee et al. [88], who stated that correctly using technology in the learning process is essential as learners need access to digital tools such as smartphones, laptops, and high-speed Internet to find SDL helpful. Other researchers applied *self-directed learning with a technology scale* (SDLTS) to a young group of students who were 10–12 years old in Singapore [89]. Considerable discussion was given to how technology use was effective in SDL, but words of caution were voiced about how the assessment was undertaken. This was due to the authors' belief that assessment tools focused on higher education students and adults rather than young learners. Anwar et al. [90] also validated the importance of self-control in Pakistani Nursing students' use of SDL. They reported that 90% of the nursing students felt that learning desire was important in their *self-directed learning*.

### 5.2 Peer learning model second-order CFA results discussion (n = 183)

The final student-teacher second-order CFA for SDL, competency from peer learning, determined that self-control (0.93) was valued most by the student-teachers. Their self-management (0.82) skills and learning desire (0.81) were second and third, respectively.

Moreover, students who take control and regulate their learning are more likely to develop intrinsic motivation, build confidence, acquire critical thinking skills, and establish social interaction while developing better help-seeking abilities [91].

Additionally, in Thailand, Ulla and Perales [92]noted the importance of the social network Facebook as a learning platform in peer learning. The authors reported that students could share their intellectual discussions and resources with classmates. The authors also suggested that given the right online pedagogical strategy, Facebook can be used as an alternative to an established learning management system, especially when a university does not have one.

### 5.3 Total group model second-order CFA results discussion (n = 468)

The final student-teacher second-order CFA for SDL competency from the total group (TG) determined that self-control (0.96) was valued most by the student-teachers, followed by their learning desire (0.80) and self-management (0.80) skills.

Early on, Dweck and Leggett noted that an individual's learning ability is their “implicit conception about the nature of ability” [93], p. 262]. The authors also noted that some students limit their ability for achievement due to their belief that they are born with limited ability and that nothing can be done to change that.

Moreover, the groups of students who learned from social media (SM), from their peers (PL), and the collective group (TG) said that they had SDL competencies in self-management, self-control, and learning desire at a high level. These assurance high levels were primarily due to the student-teachers having been using online study methods for at least two years while participating in Thailand's *New Normal* of online education during the COVID-19 outbreak. Therefore, education under the *New Normal* has caused students to develop better self-management skills, time management skills, self-discipline, planning, priorities, and self-control [94]. The students were also better at establishing their learning goals, evaluating their learning performance, and realizing their limitations. They were also able to solve problems better, design better learning processes, and encourage themselves to learn new things happily.

Finally, Ability Theory also suggests that the learning process has three pillars. This includes *self-direction, self-control*, and *self-determination*. This can be regarded as the basis for other approaches or theories. As such, learners must value these abilities to develop and fulfill them [64].

## 6 Conclusion

The research objective was to investigate and evaluate how student-teacher self-directed learning (SDL) was influenced by their peers and social media. Three models were then developed, with the *social media* (SM) model containing 285 participants, the *peer learning* (PL) model containing 183 participants, and the *total group* (TG) model containing everyone (*n* = 468) surveyed. Analysis was undertaken using second-order CFAs.

Results determined that all three aspects investigated (SM, SC, and LD) were consistent with the empirical data. Also, all three models had positive component weights (β), which differed statistically from zero at the 0.01 level. Moreover, each aspect was evaluated using eight observed variables, each showing that construct validity was good. In addition, it was found that all three aspects of each student-teachers SDL competency had high reliability (CR ≥ 0.60).

Moreover, in the Pearson Product Moment Correlation (*r*) analysis of the 24 variable relationships, the strongest was related to each student-teacher's *learning desire* (0.74). However, the weakest was *a10* to *a2* (0.16), which related to each student-teacher's inability to set high personal standards and the self-discipline to achieve them. It was also determined that the student-teachers had a high opinion about the students' SDL competency overall and in each aspect. The mean in order from highest to lowest was *self-control* (mean = 4.10), *learning desire* (mean = 4.07), and *self-management* (mean = 3.87), respectively.

Interestingly, 60.90% of the student-teachers indicated that self-directed learning is better achieved through social media than peer learning. Also, peer learning in higher education has been determined to be highly effective, but its implementation and success can be limited by which assessment process is used with it.

For this study, the authors defined the term ‘*New Normal*’ as an online educational process created to solve social distancing requirements during the global Covid-19 pandemic. However, the *New Normal* is not new but an accelerated continuation of a process that began in the early 1960s with student terminals linked through a central computer. With technology expanding at a feverish pitch since then, numerous methods (AOL to the Internet), devices (modems to smartphones), and pedagogies (student-centered learning to flipped and blended) have been created to take advantage at each stage of the technological revolution.

However, the *New Normal* has its perils and difficulties. The difficulty list is long, but it includes limits, costs, and expertise of technical support staff. There is also the ICT infrastructure cost, speed, reliability, and access issues. Additionally, training is necessary for those who must implement, use, and teach using the required hardware and software. Another problem is the fear and hesitancy of many teachers who have been forced to use unfamiliar technology with little to no training. Some are even forced to teach classes and subjects in which they have no background or qualifications. Others are also terrified at giving up traditional teaching methods where teaching is teacher 'centric’ (chalk and talk) compared to a new form of teaching where the student is put at the center of the learning process and knowledge discovery.

Finally, a debate is also raging about how students and teachers are affected when traditional classroom social interaction and support processes are eliminated. UNESCO studies have stated explicitly that a school's core mission is student well-being promotion which is best served by children learning and developing social competencies in a structured setting. Related to this is the school's role in feeding the disadvantaged and providing proper daily nutrition. The question then becomes how these needs can be met through online education under the *New Normal*. Thailand has identified school, community, and professional learning communities as a start, but solutions are needed, and they are needed quickly.

## 7 Limitations and future research suggestions

The research is limited as the research participants were from a single Thai university near Thailand's capital city Bangkok. This research was conducted in 2021 during the COVID-19 pandemic. Data collection was therefore performed using an online questionnaire. Social media was determined to be a learning medium from the research. As such, the study assures readers that if social media platforms are used correctly in learning, social media use will contribute to the continuous development of students. Future research is suggested to expand on how social media platforms can be organized and implemented around vital ICT services, state-of-the-art learning management systems (LMSs), and professional learning communities (PLCs) in higher education. A knock-on effect to this will increase student and classroom management and evaluation processes.

## Author contribution statement

Paitoon Pimdee, Ph.D.: Analyzed and interpreted the data; Contributed reagents, materials, analysis tools or data; Wrote the paper.

Attaporn Ridhikerd, Ph.D; Surapong Siripongdee, Ph.D: Conceived and designed the experiments; Performed the experiments.

Sangutai Moto, Ph.D; Suwanna Bengthong, Ph.D: Performed the experiments; Analyzed and interpreted the data; Contributed reagents, materials, analysis tools or data; Wrote the paper.

## Funding statement

This work was supported by a research grant from the School of Industrial Education and Technology, King Mongkut’s Institute of Technology Ladkrabang (2559-03-0012),Bangkok, Thailand.

## Data availability statement

Data included in article/supp. material/referenced in article.

## Declaration of interest’s statement

The authors declare no competing interests.
